# Effects of cerebral amyloid angiopathy on the brain vasculome

**DOI:** 10.1111/acel.13503

**Published:** 2022-07-18

**Authors:** Wenjun Deng, Shuzhen Guo, Susanne J. van Veluw, Zhanyang Yu, Su Jing Chan, Hajime Takase, Ken Arai, MingMing Ning, Steven M. Greenberg, Eng H. Lo, Brian J. Bacskai

**Affiliations:** ^1^ Neuroprotection Research Laboratories Department of Radiology and Neurology Massachusetts General Hospital Harvard Medical School Charlestown Massachusetts USA; ^2^ Department of Neurology Clinical Proteomics Research Center Massachusetts General Hospital Harvard Medical School Boston Massachusetts USA; ^3^ Department of Neurology J. Philip Kistler Stroke Research Center Massachusetts General Hospital Harvard Medical School Boston Massachusetts USA; ^4^ MassGeneral Institute for Neurodegenerative Disease Massachusetts General Hospital Harvard Medical School Charlestown Massachusetts USA

**Keywords:** aging, brain vasculome, cerebral amyloid angiopathy, cerebral endothelial transcriptome

## Abstract

β‐amyloid (Aβ) deposits in brain blood vessel walls underlie the vascular pathology of Alzheimer's disease (AD) and cerebral amyloid angiopathy (CAA). Growing evidence has suggested the involvement of cerebrovascular dysfunction in the initiation and progression of cognitive impairment in AD and CAA patients. Therefore, in this study, we assessed the brain vasculome in a mouse model in order to identify cerebrovascular pathways that may be involved in AD and CAA vascular pathogenesis in the context of aging. Brain endothelial cells were isolated from young and old wild‐type mice, and young and old transgenic mice expressing Swedish mutation in amyloid precursor protein and exon 9 deletion in presenilin 1 (APPswe/PSEN1dE9). Microarray profiling of these endothelial transcriptomes demonstrated that accumulation of vascular Aβ in the aging APPswe/PSEN1dE9 mouse is associated with impaired endothelial expression of neurotransmitter receptors and calcium signaling transductors, while the genes involved in cell cycle and inflammation were upregulated. These results suggest that the vascular pathology of AD and CAA may involve the disruption of neurovascular coupling, reactivation of cell cycle in quiescent endothelial cells, and enhanced inflammation. Further dissection of these endothelial mechanisms may offer opportunities to pursue therapies to ameliorate vascular dysfunction in the aging brain of AD and CAA patients.

## Graphical abstract

Vascular Aβ accumulation disrupts neurovascular coupling, induces erroneous cell cycle reactivation, and promotes inflammation in cerebral endothelium. These alterations can result in cerebrovascular dysfunction and contribute to the vascular pathology of cerebral amyloid angiopathy and Alzheimer's disease.

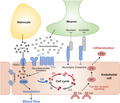

## 1 INTRODUCTION

Alzheimer's disease (AD) and cerebral amyloid angiopathy (CAA) are two major contributors to age‐related cognitive decline (Arvanitakis et al., 2011; Boyle et al., 2015; Viswanathan & Greenberg, 2011). The deposition of β‐amyloid (Aβ) peptides within cerebral blood vessels is the primary signature of CAA, and this also occurs in 85–95% of AD patients (Jellinger, 2002; Pfeifer et al., 2002). Even in the non‐demented population, the presence of vascular amyloid deposits is associated with impaired executive function and predicts future dementia risk (Case et al., 2016; Xiong et al., 2016).

Impaired cerebrovascular function is one of the earliest preclinical features in AD, preceding neuronal damage by many years (Sweeney et al., 2019). Patients at early AD stage are frequently observed with various vascular lesions such as blood–brain barrier (BBB) leakage, cerebral microbleeds, and multiple microinfarcts (Montagne et al., 2016; Poliakova et al., 2016; van de Haar et al., 2016). Cerebral blood flow deficiency resulted from impaired vascular reactivity has long been proposed as an early biomarker of AD and cognitive impairment (Bradley et al., 2002; Mazza et al., 2011). Aβ peptides demonstrate strong vasoactivity, capable of inducing vasoconstriction, attenuating vasodilation, and impairing cerebral autoregulation (Dietrich et al., 2010). Vascular Aβ buildup in CAA increases the occurrence of microbleeds and cerebral infarction (DeSimone et al., 2017; Greenberg et al., 2009). Therefore, it is possible that vascular Aβ deposition may account for the early vascular abnormalities in CAA and AD development (Peca et al., 2013; van Opstal et al., 2017) and initiate downstream cognitive decline.

As the interface between blood and brain, cerebral endothelium is the major component of BBB essential for maintaining BBB integrity. It is also a crucial regulator of vascular tone, producing various vasodilators and vasoconstrictors, such as nitric oxide (NO; Sandoo et al., 2010). Aβ peptides were found to reduce endothelial NO production through deactivating nitric oxide synthase (eNOS) and impair endothelium‐dependent vasodilation (Lamoke et al., 2015). Aβ peptides can also induce oxidative stress and activate apoptosis in cerebral endothelium, leading to endothelial damage and BBB damage (Xu et al., 2001). Notably, since cerebral endothelium is responsible for Aβ clearance via the mechanisms involving low‐density lipoprotein receptor‐related protein‐1 (LRP1) and phosphatidylcholine‐binding clathrin assembly protein (PICALM; Storck et al., 2016; Zhao et al., 2015), deregulated endothelial function caused by Aβ peptides may further enhance Aβ accumulation and accelerate disease progression. Taken together, the cerebral endothelium may play a central role in mediating vascular responses in AD and CAA (Greenberg et al., 2020) and systematic profiling of the cerebral endothelial transcriptome, i.e., the vasculome (Guo et al., 2012), should be important. In the current study, we systematically mapped and compared the brain vasculomes from young and old wild‐type mice versus young and old transgenic mice expressing mutant amyloid precursor protein (APP) and presenilin 1 (PSEN1) (APPswe/PSEN1dE9) that mimic the vascular deposition of amyloid in AD and CAA (Garcia‐Alloza et al., 2006).

## 2 RESULTS

### 2.1 Endothelial responses in APPswe/PSEN1dE9 mice

**FIGURE 1:**
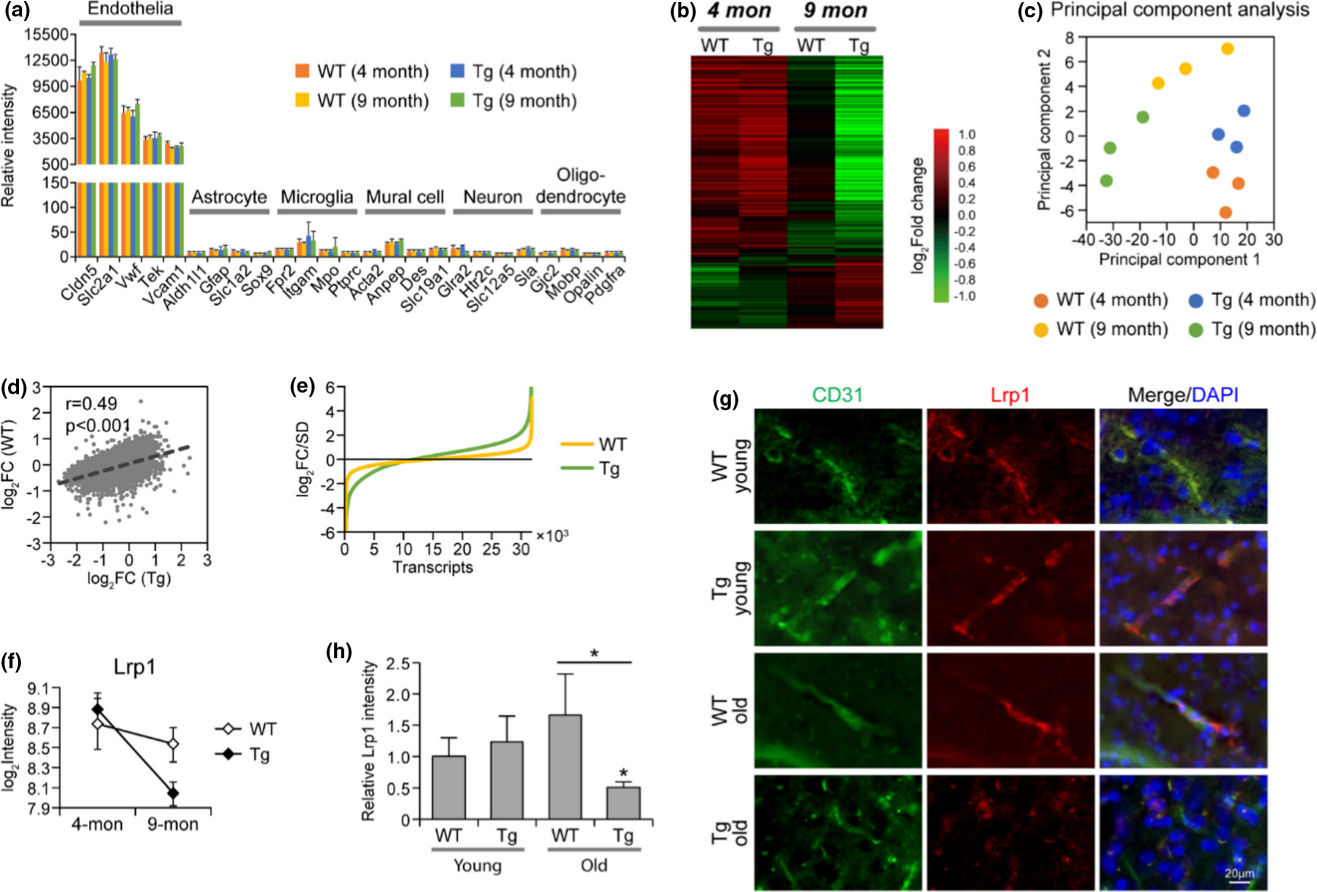
Cerebral endothelial transcriptome in CAA/AD mouse model and age‐matched WT mouse. (a) The expression of markers specific to different brain cells. Data were from three microarray samples in each group, same in the following Figures 1–5 unless otherwise specified. (b) Heatmap of differentially expressed genes in Tg and WT mouse brain endothelium. The average gene expression was determined for each group and was subjected to hierarchical clustering analysis. (c) Principal component analysis of the endothelial transcriptome in Tg and WT mice. (d) The correlation of gene expression change and (e) the extent of gene expression change between Tg and WT mouse brain endothelium. Fold change of gene expression in 9‐month‐old brain versus 4‐month‐old brain was determined respectively for Tg and WT mice. (f) Endothelial expression of Lrp1 in Tg and WT mice. (g) Immunohistochemistry staining of Lrp1 in Tg and WT mouse brain cortex, the same as used for transcriptome analysis. (h) Relative intensity of Lrp1 staining (*n* = 3 per group). Data are presented as mean ± *SD*. *p < 0.05

We previously used in vivo multiphoton microscopy to directly analyze brain blood vessels in transgenic APPswe/PSEN1dE9 mice (Tg) and found that vascular Aβ deposits in the mice begin from 6 months of age and progressively increase with age (Garcia‐Alloza et al., 2006). To investigate the early impact of vascular Aβ on cerebral vasculome, cerebral endothelial cells were isolated from Tg mice with no obvious Aβ deposition (4‐month‐old) and Tg mice with mild Aβ buildup (9‐month‐old) and were analyzed by transcriptome profiling. Endothelial cells prepared from age‐matched wild‐type mice (WT) were used as control. As shown in Figure 1a, endothelial markers, such as Cldn5, Slc2a1, Vwf, Tek, and Vcam1, were expressed at high level in all the samples, while the genes specific to other types of brain cells, such as astrocytes (Aldh1l1, Gfap, Slc1a2, Sox9), microglia (Fpr2, Itgam, MPO, Ptprc), mural cells (Acta2, Anpep, Des, Slc19a1), neurons (Glra2, Htr2c, Slc12a5, Sla), and oligodendrocytes (Gjc2, Mobp, Opalin, Pdgfra), were detected with very low abundance. These data suggest that our endothelial preparations were not contaminated with other cells.

After removing the low‐abundant genes, a total of 31,935 transcripts were identified, representing 15,318 genes. As revealed by heatmap (Figure 1b), the brain vasculome of 4‐month‐old Tg mice (i.e., before Aβ deposition) showed similar gene expression patterns to age‐matched WT mice. Differential gene expression was observed only at 9 months, the early disease stage, suggesting that endothelial dysfunction is an early event in CAA/AD development. This result was confirmed by principal component analysis, in which 4‐month‐old Tg, and WT samples were grouped together, while by 9 months, the samples from Tg and WT mice were further apart (Figure 1c). The changes induced by vascular Aβ accumulation in Tg mice is highly correlated with normal aging‐related changes in WT (Figure 1d), but occurred to a greater degree (Figure 1e), suggesting that CAA/AD progression in the brain vasculome is related to an accelerated process of aging. Of note, LRP1, a cell surface receptor‐mediating Aβ clearance, was significantly decreased in 9‐month‐old Tg mice compared with WT vasculome (Figure 1f). Immunohistochemistry staining confirmed the reduction of endothelial LRP1 in aging Tg brain but not in WT (Figure 1g,h), indicating impaired Aβ clearance in Tg mice brains.

### 2.2 Impaired neurovascular coupling with vascular Aβ accumulation

**FIGURE 2:**
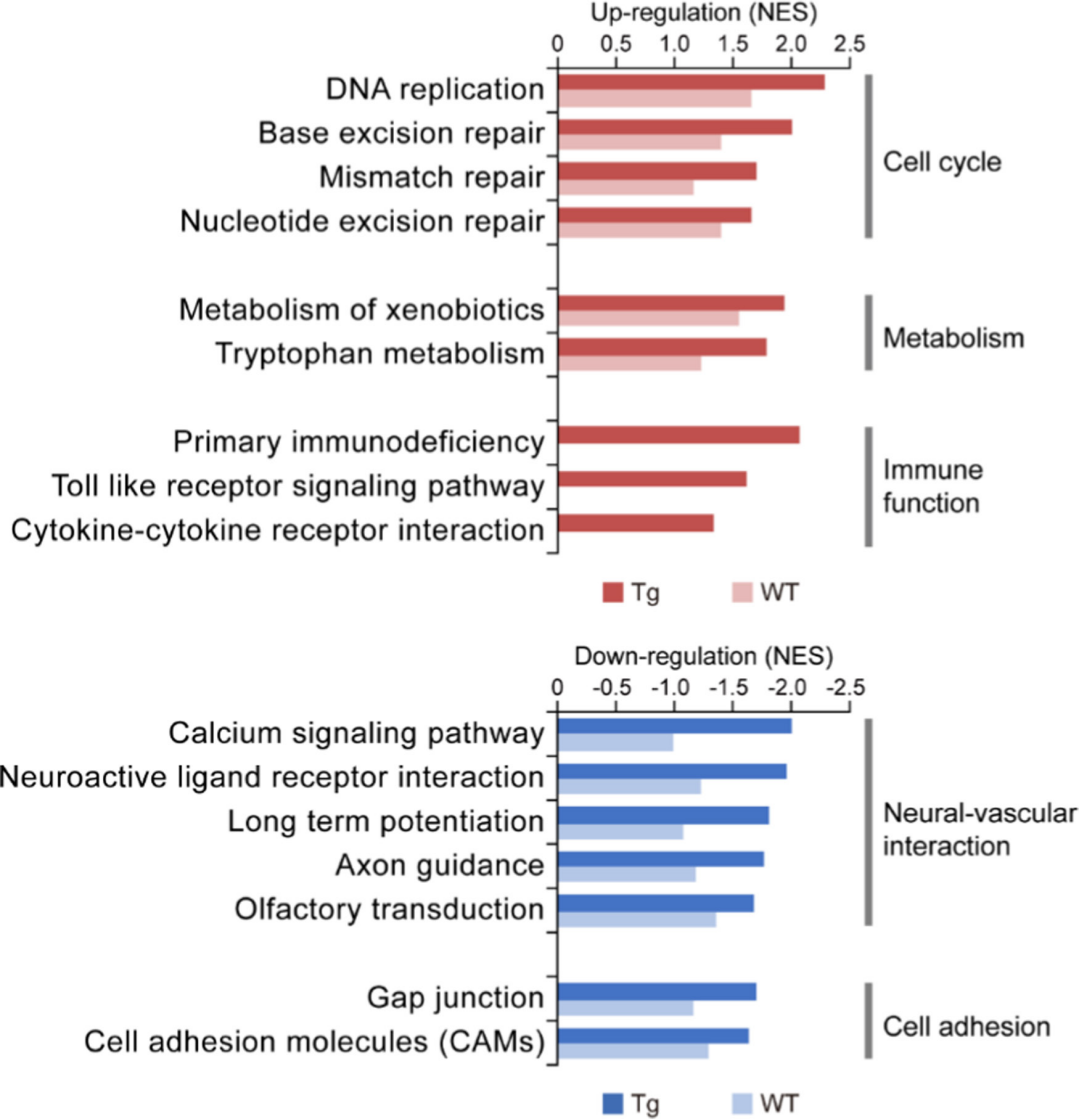
Functional alterations induced by vascular Aβ accumulation in cerebral endothelium. Genes were ranked based on differential expression in 9‐month‐old brain compared to 4‐month‐old brain for Tg and WT mice respectively. The enrichment of pathways with differentially expressed genes were analyzed by GSEA. The normalized enrichment score (NES) is shown for the most affected gene sets enriched with upregulated (red) and downregulated (blue) genes

We then performed Gene Set Enrichment Analysis (GSEA) to examine the functional vasculome changes in CAA/AD brain. As shown in Figure 2, vascular Aβ deposition in Tg mice was associated with major changes in neural–vascular interaction and cell adhesion, cell cycle, immune function, and metabolism. Similar functional alterations were also observed during the aging of WT vasculome but to a lesser extent.

**FIGURE 3:**
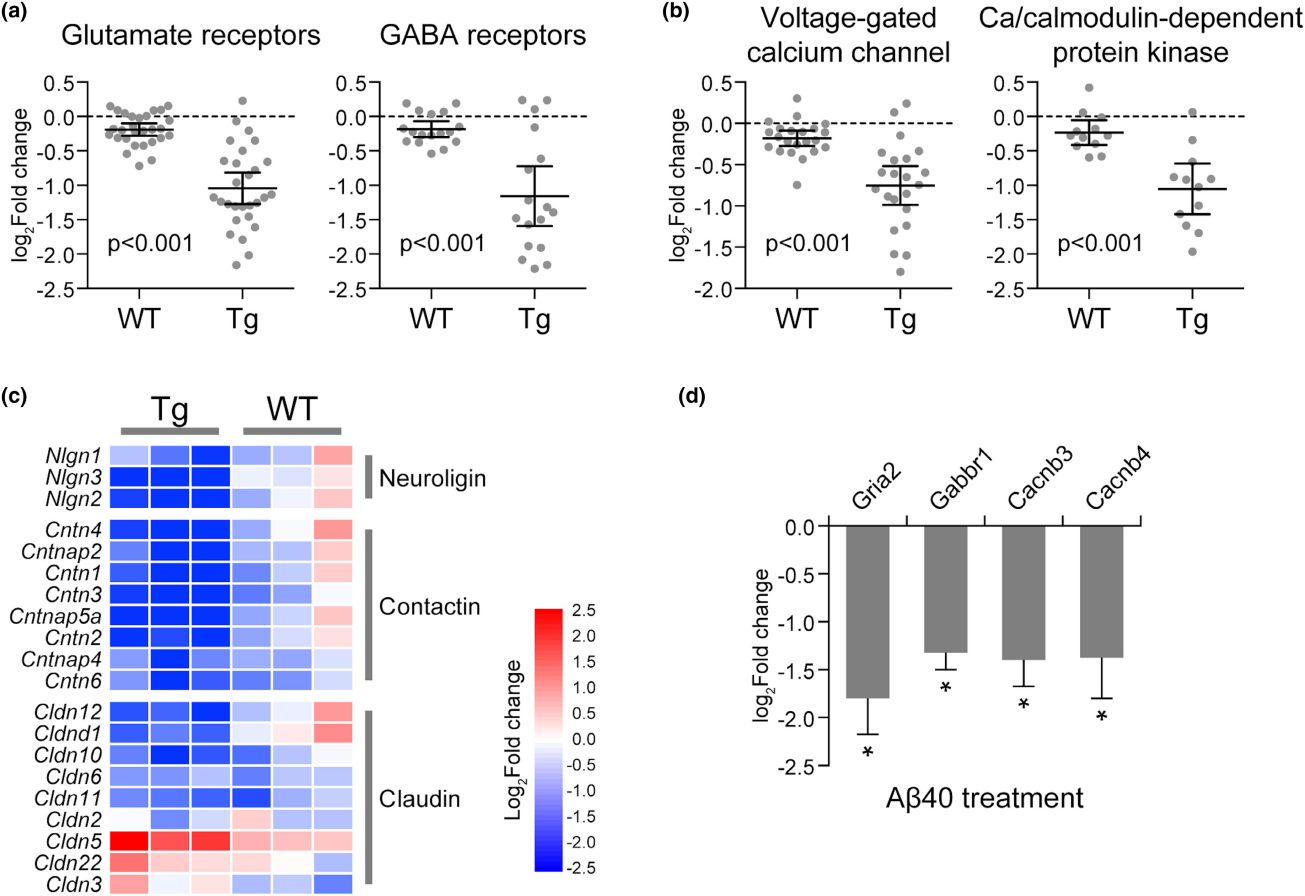
Impaired neurovascular coupling with vascular Aβ accumulation. The expression change of (a) neurotransmitter receptors and (b) voltage‐gated calcium channel and Ca/calmodulin‐dependent protein kinases in Tg and WT mouse brain endothelium. Each dot represents a specific gene. The fold change of each gene in 9‐month‐old brain versus 4‐month‐old brain was determined respectively for Tg and WT mice. (c) Heatmap of cell adhesion molecules and cell junction molecules in Tg and WT mouse brain endothelium. (d) RT‐PCR analysis of Gria2, Gabbr1, Cacnb3, and Cacnb4 expression in HBMEC treated by Aβ40 versus vehicle (*n* = 5). Data are presented as mean ± *SD*. *p < 0.05

Neuronal and vascular interaction was impaired with vascular Aβ accumulation (Figure 2). The receptors of various neurotransmitters were consistently downregulated in cerebral endothelium, such as glutamate receptors, GABA receptors (Figure 3a), indicating an impaired vascular capability of sensing neural activity. Concomitant changes were also observed for calcium signaling pathway, manifested in part as decreases of voltage‐dependent calcium channel (VDCC) and calcium/calmodulin‐dependent protein kinases (CaMKs) in the CAA/AD brain vasculome (Figure 3b). Since endothelial calcium is a major second messenger activated by neurotransmitters and participates in the production of multiple vasodilatory mediators (Guerra et al., 2018), the inactivation of calcium signaling pathway can impair endothelium‐dependent vasodilatory response to neural activation. Moreover, cell adhesion molecules regulating synaptic adhesion (neuroligins, contactins) and cell junction molecules of BBB (claudins) were also decreased in Tg mice, indicating a structural breakdown of neurovascular interface and BBB with Aβ aggregation (Figure 3c). These changes could potentially disrupt neurovascular coupling as previously reported (Peca et al., 2013). To further establish the relationship between Aβ and endothelial alterations, we directly treated human cerebral endothelial cells with Aβ40, the primary Aβ isoform in vascular amyloid depositions. We found that Aβ40 treatment led to significant reduction of genes encoding glutamate receptor subunit 2 (Gria2), GABA receptor subunit 1 (Gbbr1), and VDCC subunit beta 3 and beta 4 (Cacnb3, Cacnb4; Figure 3d), confirming a causal role of Aβ in disrupting neurovascular coupling.

### 2.3 Erroneous cell cycle reentry

**FIGURE 4:**
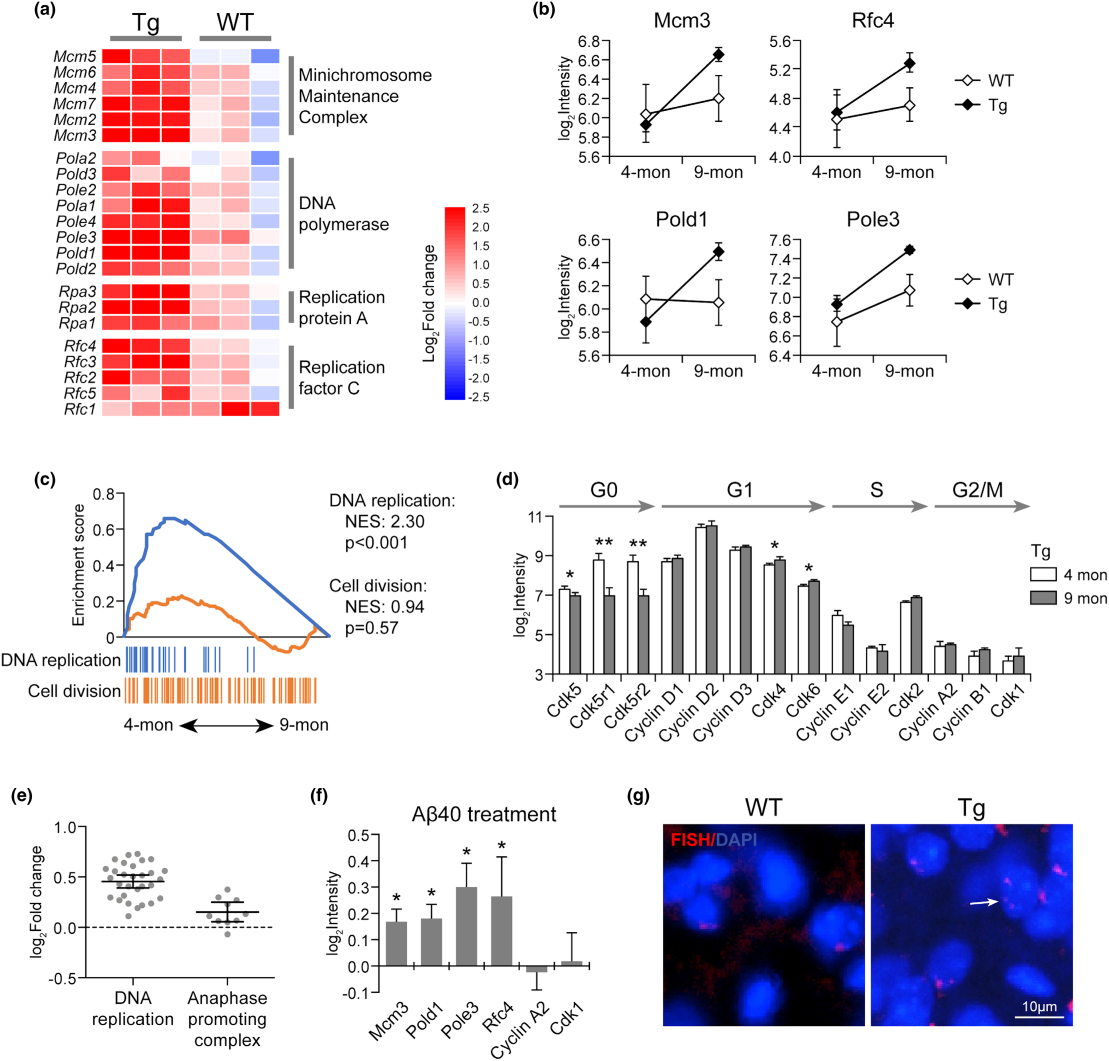
Aberrant cell cycle reentry in CAA/AD mouse brain endothelium. (a) Heatmap of DNA replication‐related genes in Tg and WT endothelium. (b) The expression of DNA replication regulators (Mcm3, Pold1, Pole3, and Rfc4) in Tg and WT mouse brain endothelium. (c) The differential activation status of DNA replication and cell division, (d) the expression change of markers at each cell cycle phase, and (e) the expression change of the genes involved in DNA replication and APC in Tg mouse brain endothelium during vascular Aβ accumulation. (f) RT‐PCR analysis of cell cycle‐related genes in HBMEC treated by Aβ40 versus vehicle (*n* = 5). (g) FISH assay of polyploidy in Tg and WT brain sections at 9‐month‐old. Data are presented as mean ± *SD*. *p < 0.05; **p < 0.01

Pathway analysis identified the activation of cell cycle progression in cerebral endothelium of Tg mice (Figure 2). As shown in Figure 4a–c, DNA replication was significantly enhanced with vascular Aβ accumulation in Tg brain. The subunits of many complexes involved in DNA synthesis, such as minichromosome maintenance complex (e.g., Mcm3), DNA polymerase complex (e.g., Pold1, Pole3), replication protein A family, and replication factor C family (e.g., Rfc4), were significantly upregulated (Figure 4a,b), indicating cell cycle reactivation in quiescent endothelial cells. This result was further supported by the downregulation of G0 phase markers, Cdk5, Cdk5r1, and Cdk5r2 (Cicero & Herrup, 2005; Zhang et al., 2010) and the upregulation of G1 phase markers, Cdk4 and Cdk6 (Figure 4d). However, we did not see a coordinated activation of genes for cell division (Figure 4c). The regulators of G2/M transition, cyclin A, cyclin B, and Cdk1 were all expressed at very low levels and showed no dramatic expression change (Figure 4d). The anaphase‐promoting complex (APC) regulating sister chromatin segregation in M phase was not upregulated either (Figure 4e), suggesting the cells are arrested at some point before M phase. These results were further verified by in vitro treatment of endothelial cells with Aβ40. As shown in Figure 4f, genes involved in DNA replication, including Mcm3, Pold1, Pole3, and Rfc4, were upregulated by Aβ40 treatment, while no changes were observed for M phase markers, cyclin A2 and Cdk1. The mismatch between DNA duplication and cell division can generate polyploid cells. As revealed by fluorescence in situ hybridization (FISH), polyploidy was detected in aging Tg brain endothelium rather than in WT brain (Figure 4g), which may lead to genomic instability and added endothelial dysfunction.

### 2.4 Enhanced inflammation associated with vascular Aβ accumulation

**FIGURE 5:**
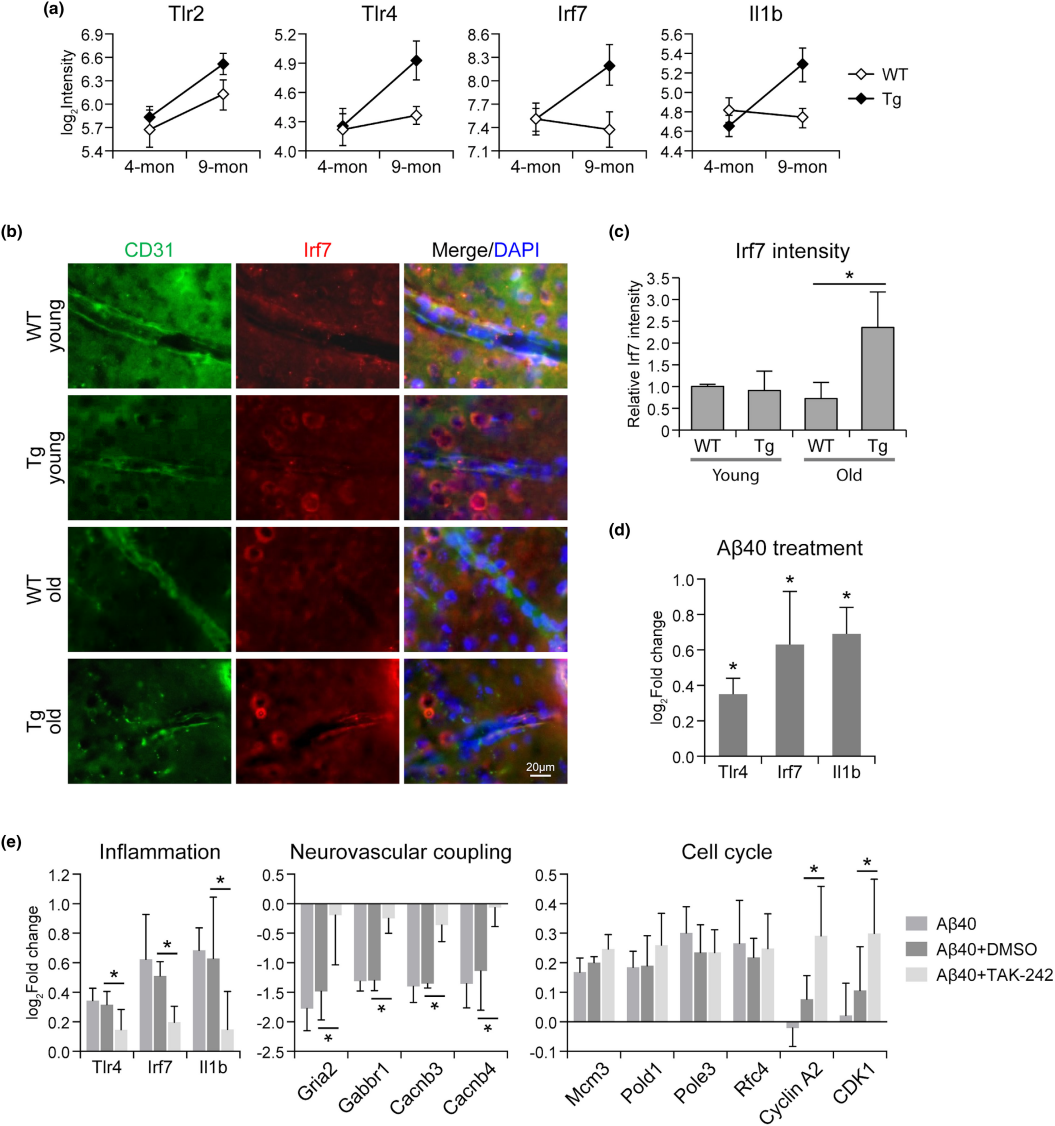
Enhanced vascular inflammation in CAA/AD mouse brain endothelium. (a) The expression of genes involved in toll‐like receptor signaling pathway in Tg and WT mouse brain endothelium. (b) Immunohistochemistry staining of Irf7 in Tg and WT mouse brain cortex, the same area used for endothelium transcriptome analysis. (c) Relative intensity of Irf7 staining (*n* = 3 per group). (d) RT‐PCR analysis of inflammation genes in HBMEC treated by Aβ40 versus vehicle (*n* = 5). (e) TLR4 inhibitor TAK‐242 reversed the gene expression changes induced by Aβ40 in HBMEC. Data are presented as mean ± *SD*. *p < 0.05

Vascular Aβ accumulation in Tg mouse was associated with enhanced inflammation (Figure 2). Genes involved in toll‐like receptor (TLR) signaling pathway and cytokine–cytokine receptor interaction, such as Tlr2, Tlr4, Irf7, and Il1b, were upregulated in the endothelium of 9‐month‐old Tg mice but not in WT (Figure 5a). The elevation of endothelial Irf7 in aging Tg mice was further validated by immunostaining of brain tissue (Figure 5b,c). In an in vitro endothelial cell culture model, upregulation of Tlr4, Irf7, and Il1b was observed after Aβ40 treatment (Figure 5d). Inhibiting TLR4 with TAK‐242 could decrease the inflammatory factors (Tlr4, Irf7, Il1b) upregulated by Aβ40, and also reverse the downregulation of neurotransmitter receptors and calcium channel subunits (Gria2, Gbbr1, Cacnb3, Cacnb4; Figure 5e). Moreover, TAK‐242 treatment also induced the expression of M phase regulators (cyclin A2, Cdk1) that failed to be activated by Aβ40, which could promote cell cycle progression and maintain genome stability (Figure 5e). We thus concluded that endothelial Aβ accumulation may contribute to a vascular proinflammatory status while inhibiting TLR4‐mediated inflammation could reverse Aβ‐induced alterations and serve as a potential therapeutic target for CAA/AD treatment.

### 2.5 Comparison of CAA/AD vasculome with other brain disorders

Analysis of the brain vasculome in Tg mice identified alterations in neurovascular uncoupling, cell cycle, and inflammation during the early development of CAA/AD. Because many other brain disorders are also known to damage cerebral endothelium and impair cognition, we compared our findings with prior cerebral endothelial studies in hypertension, diabetes, traumatic brain injury (TBI), epilepsy, stroke, and experimental autoimmune encephalomyelitis (EAE) (Guo et al., 2019; Munji et al., 2019).

**FIGURE 6:**
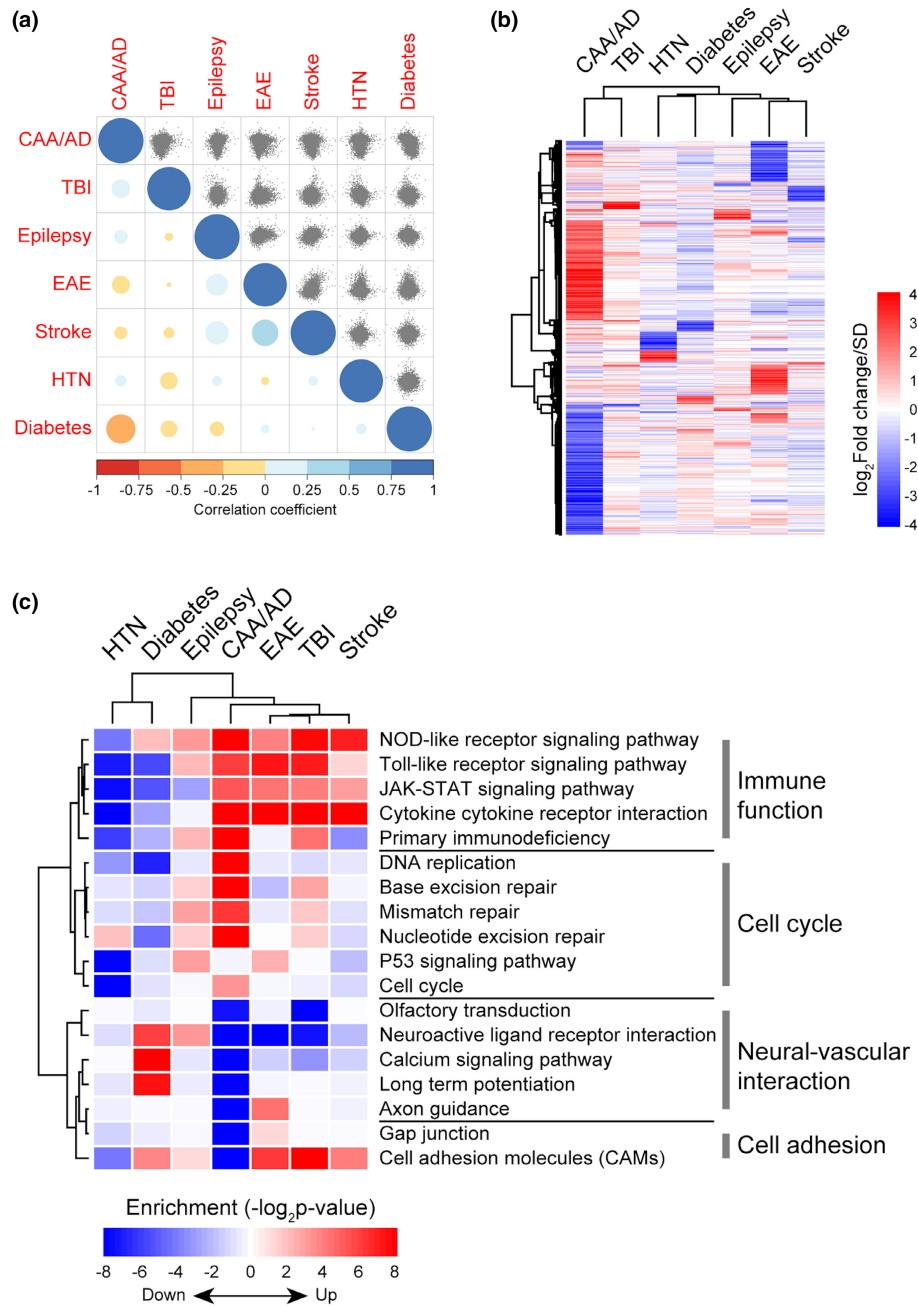
Comparison of cerebral endothelial alterations in various brain disorders. (a) The brain endothelial gene expression correlation across various disease conditions. The correlation of endothelial gene expression change between pairs of conditions was presented as scatter plots in the upper triangle matrix. The Pearson correlation coefficients were presented by the color and size of each dot in the lower triangle matrix. (b) Hierarchical clustering of significantly changed endothelial genes (fold change >1.2; FDR <0.05) in each disease condition. (c) Functional alterations in brain endothelium of each disease condition. GSEA was performed on genes preranked according to their expression changes in each disease condition compared with corresponding control. HTN: hypertension; TBI: traumatic brain injury; EAE: experimental autoimmune encephalomyelitis. HTN and diabetes data were obtained from (Guo et al., 2019); TBI, epilepsy, stroke, and EAE data were obtained from GSE95401 (Munji et al., 2019). For CAA/AD, HTN, diabetes, epilepsy, stroke, and EAE data, *n* = 3 per group; for TBI data, *n* = 3 for TBI group, *n* = 2 for control group

Although all these conditions are risk factors of cognitive decline, no obvious correlation of endothelial gene expression were detected (Figure 6a) and significantly changed genes showed little overlap among the various diseases (Figure 6b), suggesting that each disease induces a distinctive gene expression pattern. However, when we explored the pathway alterations caused by each disease (Figure 6c), we found that immune functions, such as toll‐like receptor signaling pathway and cytokine–cytokine receptor interaction, were activated in CAA/AD, EAE, TBI, and stroke. Neurovascular interaction, particularly neuroactive ligand–receptor interaction, was inhibited in CAA/AD, EAE, and TBI. All these suggested that enhanced inflammation and impaired neurovascular coupling are common endothelial changes in brain disorders. In contrast, cell cycle process was more activated in CAA/AD than in other disease conditions, indicating a potentially unique importance of cell cycle reentry in CAA/AD pathogenesis.

### 2.6 Endothelial cell cycle reentry was conserved in AD patients

**FIGURE 7:**
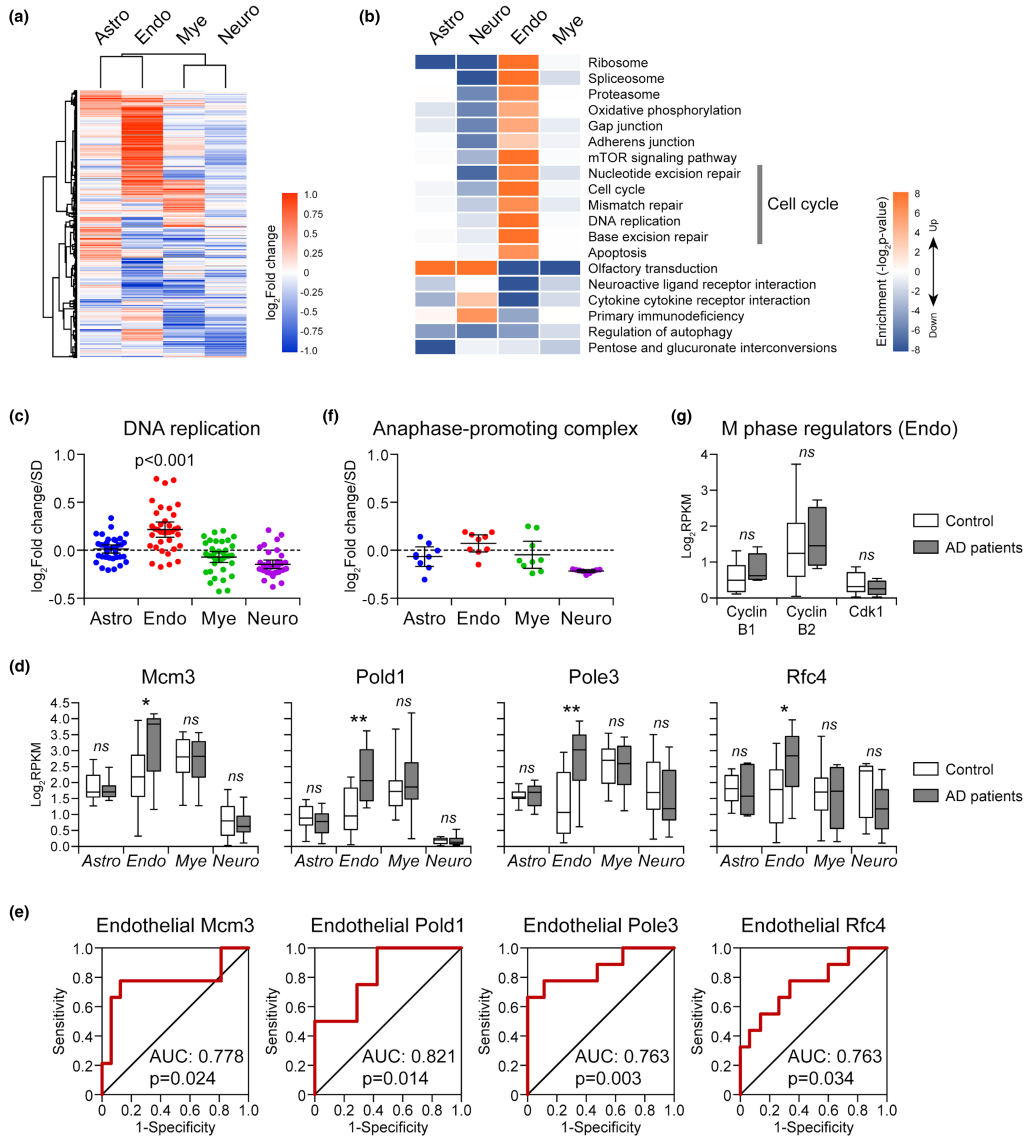
Cell cycle activation status in different brain cells of AD patients. (a) Heatmap of differentially expressed genes in different brain cells of AD patients. (b) Functional alterations in different brain cells with AD development. (c) The expression changes of genes involved in DNA replication in different brain cells of AD patients. (d) The expression of Mcm3, Pold1, Pole3, and Rfc4 in different brain cells of AD patients. (e) Receiver–operator characteristic (ROC) curves for DNA replication regulators in brain endothelium. (f) The expression changes of genes involved in APC in different brain cells of AD patients. (g) The expression change of M phase regulators in AD endothelium. The expression data of AD patients used here were obtained from GSE125050 (Srinivasan et al., 2020). Astro: astrocyte; Neuro: Neuron; Endo: endothelial cell; Mye: myeloid. For Astro, *n* = 7 (AD group), *n* = 12 (control group); for Endo, *n* = 10 (AD group), *n* = 17 (control group); for Mye, *n* = 10 (AD group), *n* = 15 (control group); for Neuro, *n* = 21 (AD group), *n* = 21 (control group). *p < 0.05; **p < 0.01; ns, not significant in AD versus control

Finally, we compared our results with human AD data in different types of brain cells, including endothelial cells, astrocytes, neurons, and myeloid cells (GSE125050; Srinivasan et al., 2020). As shown in Figure 7a, different brain cells demonstrated different changing pattern with AD development. Consistent with the changes in CAA/AD mice brain endothelium, human AD brain endothelium also showed aberrant cell cycle reentry (Figure 7b). Genes involved in DNA replication (Mcm3, Pold1, Pole3, Rfc4) were upregulated in human AD endothelium (Figure 7c,d) and were able to discriminate AD patients with high accuracy (Figure 7e). Moreover, no significant change was found for M phase regulators, such as cyclin B, CDK1, and APC, suggesting that AD endothelial cells may likewise be arrested at some point before division (Figure 7f,g). Cell cycle reentry has been reported in AD neurons and considered as an early feature of AD (Bonda et al., 2010; Lee et al., 2009). However, according to our analysis of different cell types in AD brain, cell cycle activation was mostly observed in cerebral endothelium but not in neurons or other brain cells (Figure 7b–d), suggesting that cell cycle abnormality in cerebral endothelium might in fact, be a more common phenomenon in AD patients.

## 3 DISCUSSION

**FIGURE 8:**
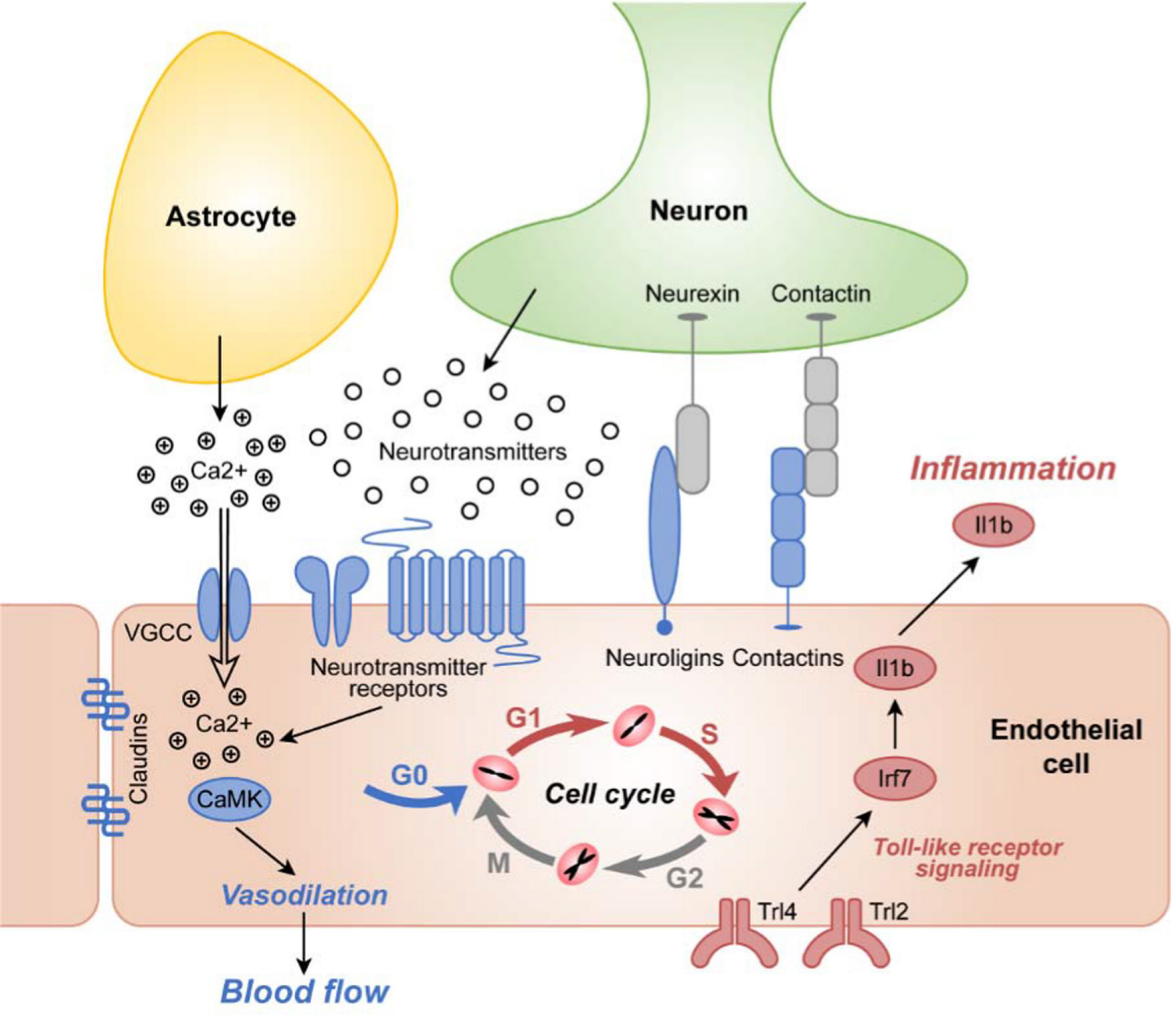
A schematic overview of cerebral endothelial alterations in the context of CAA. Vascular Aβ accumulation is associated with aberrant cell cycle activation, disrupted neurovascular coupling, and enhanced inflammation in cerebral endothelium. Red color: upregulation; blue color: downregulation

In the present study, we systematically profiled the cerebral vasculome in a CAA/AD mouse model and found that even in the presence of mild vascular Aβ accumulation, cerebral endothelium demonstrates cell cycle abnormality, neurovascular uncoupling, and enhanced inflammation (Figure 8), suggesting that these processes might play a role in early disease pathogenesis.

Neuronal cell cycle reentry without cell division is an early brain change in AD patients (Bonda et al., 2010; Lee et al., 2009). Many G1 and S phase regulators are re‐expressed in postmitotic neurons vulnerable to degeneration, including cyclin D‐CDK4/CDK6 complex responsible for initiating G1 phase, cyclin E‐CDK2 complex controlling the transition from G1 to S phase, and minichromosome maintenance protein MCM2 regulating DNA replication in S phase (Bonda et al., 2009; McShea et al., 1997; Nagy et al., 1997; Vincent et al., 1997). However, since no evidence has shown the activation of M phase markers and no actual cell division has ever been observed, the reactivation of cell cycle in AD neuron usually generates polyploid neurons, leading to dysregulated neuronal function and neuronal death culminating in neurodegeneration (Mosch et al., 2007; Zhu et al., 2008).

Oxidative stress‐induced DNA damage and repair has been considered a major cause of cell cycle activation in AD neurons. In addition, Aβ also has mitogenic activity and is able to activate the kinases involved in tau phosphorylation (fyn, PKA and CaMKII) and trigger cell cycle reentry via a tau‐mediated mechanism (Seward et al., 2013). In our study, we found that when exposed to vascular Aβ, cerebral endothelium also underwent erroneous cell cycle reentry. The decrease of G0 phase markers (CDK5, CDK5r1, CDK5r2) and the increase of G1 phase markers (CDK4, CDK6) suggested the exit from G0 phase and the reentry into cell cycle. The significant activation of DNA replication further confirmed the initiation and completion of G1 and S phases. Moreover, similar to AD neurons, no activation of M phase markers was detected, which may as well induce genomic instability in endothelial cells and leave the cells more susceptible to apoptosis. Since proper endothelial function is the basis of BBB integrity, endothelial dysfunction due to the aberrant cell cycle may disrupt BBB and expose neurons to the neurotoxic factors circulating in the blood. In normal condition, mature endothelial cells are trying to stay long in a quiescent state despite their ability of self‐renewal (Rajendran et al., 2013; Schlereth et al., 2018). Disruption of this phenotype is usually associated with a wide range of diseases, such as sepsis, atherosclerosis, and cancer. According to our results, loss of endothelial quiescence could also be an early feature of vascular cognitive decline. Since the animal used here was at the early stage of CAA, it is very likely that ectopic cell cycle events in cerebral endothelium may occur earlier than that in neurons or even be a causal event of neural dysfunction. More importantly, we found that in AD patients, aberrant cell cycle was more common in cerebral endothelium than in neuron and other brain cells, and could thus be an early therapeutic target benefiting more patients at high risk of dementia.

Erroneous cell cycle reentry can cause a variety of cellular dysfunction before proceeding toward apoptosis. In our study, endothelial dysfunction was manifested as impaired neurovascular coupling and enhanced inflammation. Neurovascular coupling is a mechanism whereby the local cerebral blood flow is adjusted in response to the changes in neuronal activity, while disruption of this process may cause a mismatch between energy supply and demand and lead to subsequent brain dysfunction (Iadecola, 2017). Of note, cerebral blood flow defect as a result of neurovascular uncoupling has been detected in the CAA/AD mice model used in our study (Kara et al., 2012), and is frequently associated with the early progression of AD, indicating a causal role of neurovascular uncoupling in cognitive decline (Nortley et al., 2019; Stefanovic et al., 2008). Although the endothelial function has been well characterized in peripheral vasculature, the involvement of cerebral endothelium in neurovascular coupling remained barely known to us (Guerra et al., 2018). Despite this, as observed in our and others’ studies (Krizbai et al., 1998; Li et al., 2018), the extensive expression of neurotransmitter receptors and the cell adhesion molecules that are widely expressed in postsynaptic membrane (neuroligins, contactins) implies a functional and structural interaction between endothelium and neural activity. In capillary, the most numerous blood vessel in the brain, endothelial cells are of particular importance in initiating neurovascular coupling and propagating dilation signals into upstream arterioles, as they are the only component of capillary vessel wall (Hamilton et al., 2010; Zlokovic, 2005). All the evidence suggested that impaired endothelial response to neural activity during CAA/AD development, as observed in our study, might be among the earliest events leading to blood flow reduction and consequent cognitive decline.

In addition to impaired neurovascular coupling, we also revealed that enhanced inflammation in cerebral endothelium may also be an early feature associated with vascular Aβ accumulation. This is fully consistent with the recent report that endothelial cells contribute to the immune response in AD pathogenesis (Lau et al., 2020). The activation of proinflammatory TLR signaling pathway in endothelial cells recruits more neutrophil to brain capillary, which further worsens blood flow deficiency and promotes disease progression (Bernardes‐Silva et al., 2001; Zhou et al., 2009). Our results showed that inhibiting endothelial TLR4 signaling pathway could not only suppress the inflammation activated by Aβ but also reverse other Aβ‐induced alterations, indicating the possibility of inflammatory pathway as the target for CAA/AD treatment.

There are several limitations in the present study. First, in addition to vascular amyloid deposits, the mouse model used here also shows extensive parenchymal Aβ plaques. Although we showed evidence that the amyloid species in CAA, Aβ40 is causally associated with the endothelial alterations observed, the generalizability of our findings should be validated in other models with more severe CAA, such as the SwDI and the APP23 mouse models. Second, our study used in vitro model to identify Aβ as a cause of cerebral endothelial alterations, and identified TLR4 signaling as a target to reverse Aβ‐induced changes. Future in vivo gain and loss‐of‐function studies are required to establish the relationship between endothelial alterations and cognitive outcome in animal models, and evaluate the clinical value of cerebral endothelium for CAA/AD diagnosis and treatment. Third, the present study focused on the early stage of vascular Aβ accumulation. While understanding the initiation of CAA/AD is crucial for disease prevention, prolonged monitoring of endothelial alterations may offer a broader view of how brain vasculome dynamics interact with disease progression. Fourth, our gain and loss‐of‐function experiments in cell culture hint at but do not unequivocally prove causality. The fact that blocking TLR4 signaling appears to ameliorate Aβ‐induced perturbations in neurotransmitter receptor, calcium signaling, and cell cycle regulator genes, suggest that inflammation may be upstream of or at least connected to these functional responses in the AD or CAA vasculome. However, we acknowledge that further in vivo studies are required to dissect the mechanisms rigorously. Finally, in addition to understanding endothelial alterations, future studies are warranted to explore the responses in other cells of the neurovascular unit (e.g., pericytes, smooth muscle cells, astrocytes).

In summary, this study provides a comprehensive database of the brain vasculome in aging APPswe/PSEN1dE9 mice. Further investigations are warranted to ask whether the early endothelial alterations identified here may serve as potential therapeutic targets for CAA and AD.

## 4 EXPERIMENTAL PROCEDURES

### 4.1 Animal

The transgenic mouse line APPswe/PSEN1dE9 expressing the Swedish mutation of APP and deletion of Exon 9 in PSEN1, under the promoter of prion (Garcia‐Alloza et al., 2006), was bred and aged in house and were used at the age of 4 and 9 months. The age‐matched nontransgenic littermates were used as controls. All mice used in the study were female and housed in filtered cage, with free access to standard pellet food and water ad libitum, within temperature‐controlled room on 12 h light/dark cycle. All animal procedures were conducted in accordance with the Massachusetts General Hospital Animal Care and Use Committee and consistent with the National Institute of Health guidelines for the Care and Use of Laboratory Animals.

### 4.2 Preparation of mouse brain endothelial cells

The transgenic mouse used here was observed with Aβ deposition in both hippocampus and cortex (Garcia‐Alloza et al., 2006). Due to the small amount of hippocampus tissue, we extracted endothelial cells from the brain cortex as in our previous report (Guo et al., 2012). Briefly, mice were anesthetized by isoflurane and perfused with 40 ml phosphate‐buffered saline (PBS), and the cerebral cortex was dissected and minced. Cortexes from three mice were pooled and incubated in 2 mg/ml Collagenase/Dispase (Roche) at 37°C for 40 min with mixing every 10 min, then mechanically dissociated by titrating through 14 gauge needles. The digestions of cortex were filtered through a 70 µM cell strainer (Becton Dickinson Labware) and centrifuged at 500 ***g*** for 5 min at 4°C to get cell pellets. The raw cell pellets were then resuspended in cold HBSS (without Ca^2+^/Mg^2+^) and incubated with anti‐PECAM 1 antibody‐coated Dynabeads (Invitrogen), with gentle rotation for 30 min at 4°C. The PECAM1‐positive endothelial cells were recovered and washed with magnetic separator in HBSS for three times, then used for the RNA preparation with RNeasy Micro Plus kit (Qiagen).

### 4.3 Transcriptome profiling and functional analysis

Three RNA samples for each group were used for transcriptome study with Affymetrix GeneChip Mouse 430 2.0 arrays, provided by Microarray and Sequencing Resource Core Facility of Boston University School of Medicine. Each RNA sample was prepared from pooled cerebral cortex of three mice. All samples had RNA integrity number scores larger than 7.0 as measured on Agilent Bioanalyzer 2001. All microarray hybridization and scanning were performed after amplification with the NuGEN Ovation WTA Pico kit and fragmentation and labeling with Encore Biotin Module. The raw data for each chip were collected and normalized using RMA algorithm. The probes with average intensity less than 15 were not included in following analysis. All the identified genes were included for functional enrichment analysis with GSEA tool. The function annotation of genes was obtained from Kyoto Encyclopedia of Genes and Genomics database.

### 4.4 Public transcriptome database

Publicly available endothelial transcriptome in different disease conditions (GSE95401) and the transcriptome of different brain cells in AD patients (GSE125050) were obtained from Gene Expression Omnibus (GEO) database and were subjected to functional analysis using GSEA.

### 4.5 Immunohistochemistry staining

Coronal brain sections (40 µm) of WT mice or APPswe/PSEN1dE9 mice were fixed in 4% PFA and blocked with 5% fetal bovine serum (FBS) plus 2% goat serum in PBS for 1 h, followed by incubation at 4°C overnight with primary antibodies. The sections were then washed and incubated for 1 h with corresponding fluorescence‐conjugated secondary antibodies (Jackson ImmunoResearch). Vectashield mounting medium containing DAPI (Vector Laboratory) was used to coverslip the slides. Fluorescent signals were examined using Nikon Eclipse T300 fluorescence microscope. The primary monoclonal antibodies used here include anti‐CD31 (Novus Biological, NB600‐1475, 1:100 dilution), anti‐LPR1 (Santa Cruz Biotech, sc‐57353, 1:50 dilution), and anti‐Irf7 (Santa Cruz Biotech, sc‐74471, 1:50 dilution).

### 4.6 Fluorescent in‐situ hybridization (FISH)

FISH was performed using a Texas red‐conjugated chromosome 17 probe (Empire Genomics, CHR17‐10‐RE) following the manufacturer's instructions. Briefly, the slides were fixed for 45 min using Histochoice (AMRESCO, Solon, OH) and then dehydrated in ethanol. The probe mixture (2 µl of probe and 8 μl of buffer) was added onto the section and covered with a cover glass, which was further sealed with rubber cement. The slide set was first placed in 83°C warmer for 3 m for probe denaturation, then was placed in a humidified chamber and incubated at 37°C for 16 h. After incubation, the slide was removed from the humidified chamber, and the cover glass was removed. The slide was washed in wash solution 1 (WS1, 0.3% Igepal, Sigma CA‐630) at 73°C for 2 m, then washed in WS2 (0.1% Igepal) at room temperature for 2 m. After the slide was air dried in dark, mounting medium containing DAPI was added onto the section. The slides were examined under a Nikon Eclipse T300 fluorescence microscope.

### 4.7 Cell culture and treatment

Human brain microvascular endothelial cells (HBMEC) from Cell System Corporation were used to study the effects of amyloid β fragments. The cells were maintained with EndoGRO‐basal media (Millipore) plus EndoGRO MV‐VEGF supplement kit (Millipore), on human fibronectin (Sigma) coated culture dish. Amyloid β protein fragment 1–40 (Aβ40, Sigma, A1075) was dissolved with 1% acetic acid and then incubated at 37°C for 4 days before use. TAK‐242 (Sigma), a cell‐permeable cyclohexenecarboxylate that disrupts TLR4 interaction with adaptor molecules by selectively binding to TLR4 intracellular residue Cys747, was dissolved with DMSO. For treatment, HBMEC before passage 15 were seeded in 12‐well plates precoated with human fibronectin. Before confluent, HBMEC were exposed to Aβ40 (1 µM) for 3 days, without or with TAK‐242 (2 µM). Total RNAs were prepared from cells after treatment with RNeasy Plus Mini kit (Qiagen, 74134). Corresponding cDNA were synthesized by QuantiTeck reverse transcription kit (Qiagen, 205311). The expressions of related genes were measured with Taqman primers (Applied Biosystems) on QuantStudio 3.0, using 2^−ΔΔCt^ method.

## CONFLICT OF INTEREST

None.

## AUTHOR CONTRIBUTIONS

E.H.L. and B.J.B. designed and supervised the study and experiments; W.D., S.G., and Z.Y. performed the experiments and analyzed the data; S.J.C. and H.T. also performed the experiment; S.J.V contributed to materials; W.D. and S.G. wrote the manuscript; E.H.L., B.J.B., S.M.G., S.J.V., K.A. and M.N. reviewed the manuscript; All authors read and approved the final manuscript.

## DATASET CITATION

Daneman R, Munji R, Soung A; 2017; GEO; GSE95401.

Friedman B, Hansen D; 2019; Gene Expression Omnibus; GSE125050.

## Data Availability

The data that support the findings of this study are available from the corresponding author upon reasonable request.
